# The complexity of porphyrin-like pigments in a marine annelid sheds new light on haem metabolism in aquatic invertebrates

**DOI:** 10.1038/s41598-019-49433-1

**Published:** 2019-09-10

**Authors:** C. Martins, A. P. Rodrigo, L. Cabrita, P. Henriques, A. J. Parola, P. M. Costa

**Affiliations:** 1 UCIBIO –Applied Molecular Biosciences Unit, Departamento de Ciências da Vida, Faculdade de Ciências e Tecnologia da Universidade Nova de Lisboa, 2829-516 Caparica, Portugal; 2 LAQV –Associate Laboratory for Green Chemistry, Departamento de Química, Faculdade de Ciências e Tecnologia da Universidade Nova de Lisboa, 2829-516 Caparica, Portugal; 3 Instituto de Anatomia, Faculdade de Medicina, Universidade de Lisboa, 1649-028 Lisboa, Portugal

**Keywords:** Ecophysiology, Animal physiology

## Abstract

True green pigments in the animal kingdom are scarce and are almost invariably porphyrinoids. Endogenous porphyrins resulting from the breakdown of haem are usually known as “bile pigments”. The pigmentation of intertidal Polychaeta has long gained attention due to its variety and vivid patterning that often seems incompatible with camouflage, as it occurs with *Eulalia viridis*, one of the few truly green Polychaeta. The present study combined UV and bright-field microscopy with HPLC to address the presence and distribution of pigments in several organs. The results showed two major types of porphyrin-like pigments, yellowish and greenish in colour, that are chiefly stored as intraplasmatic granules. Whereas the proboscis holds yellow pigments, the skin harbours both types in highly specialised cells. In their turn, oocytes and intestine have mostly green pigments. Despite some inter-individual variation, the pigments tend to be stable after prolonged storage at −20 °C, which has important implications for future studies. The results show that, in a foraging predator of the intertidal where melanins are circumscribed to lining the nervous system, porphyrinoid pigments have a key role in protection against UV light, in sensing and even as chemical defence against foulants and predators, which represents a remarkable adaptive feature.

## Introduction

Most animals owe their green colour to complex chromatophores that filter visible light sequentially, rather than through biosynthesis of green pigments or their incorporation from photosynthetic organisms. The few exceptions are bile pigments, especially biliverdin, a blue-green pigment that results from the breakdown of haem groups of respiratory pigments^1,2^. Interestingly, the first animals to be described as having true green pigments were marine organisms, more specifically, Echiurans^3^. There have been several works reporting on the potential origins of green pigments in marine invertebrates and their classification according to their source^4,5^. The parent compounds of exogenous green pigments are primarily acquired from food, such as chlorophylls and their derivates, whereas endogenous pigments are mostly derived from the catalysis and transformation of endogenous haem, as biliverdin. Two important examples of each case are described in the Polychaeta: the exogenous chaetopterin identified in *Chaetopterus variopedatus* mid-gut as a chlorophyll derivative resulting from detritivore feeding^6^, and the endogenous biliverdins, such as those identified in *Hediste diversicolor*^7^. Regardless of their source, these green pigments share their tetrapyrrolic nature.

Tetrapyrroles are chemical compounds that have four pyrrole or pyrrole-like rings. This group includes not only porphyrins, like haem, chlorophylls and bile pigments, but also the closely-related chlorins and bacteriochlorins^8^. However, the classification of green tetrapyrrolic pigments from animals may not be entirely consensual or their origins easy to identify. For instance, the chlorin named bonellin from the Polychaeta (Echiura) *Bonellia viridis* was originally believed to be a derivative from chlorophyll, but later concluded that it is, in fact, an endogenous, unique chlorin^9^.

Tetrapyrrolic compounds can have a variety of functions, depending on chemical structure and modifications. Porphyrins, in particular, have a characteristic absorption spectrum with maxima in both visible and ultraviolet (UV) regions^10^. Despite their structural diversity, their absorbance spectra are characterised by the presence of Soret- and Q-bands, between 380–500 nm and 500–700 nm, respectively^11^. Besides the role of haem porphyrins in gas exchange, inclusively in invertebrates^5^, their ability to absorb UV light can be particularly relevant to intertidal marine organisms as it may confer protection from sunlight, but this ecological role of pigments is not really explored. It must be noted that there is a greater diversity of haem-bearing respiratory pigments in marine invertebrates than in vertebrates, in the Polychaeta included, who possess a Fe-containing pigment termed chlorocruorin, either as substitute or complement to haemoglobin^4^. Bile pigments are porphyrins resulting from the catabolism and recycling of these haem proteins. They play an important role in cells as antioxidants^12^ and can even hold important antimicrobial properties^13^. Nonetheless, the chemistry and underlying biosynthetic processes of haem-derived pigments in Polychaeta are not well understood. Still, the production of biliverdin and related pigments is already described and, in some cases, accompanied by rather unique adaptations, such as the ability to be stored and transported by coelomocytes in an association to glutathione and vitellogenins, as in case of *Nereis virens*, known nowadays as *Alitta virens*^14^.

The reasons mentioned above contribute for rendering porphyrins great interest for biotechnological applications. These include, for instance, as photosensitisers in photodynamic therapy of skin cancer and other diseases, due to the potential ability to generate cytotoxic oxidative radicals when activated by light^10,15^. Likely due to their ability to mediate redox reactions, the effects of porphyrins can be diverse or even seemingly paradoxical, which can be linked, e.g., to photoactivation. As an example, free porphyrins like some bile pigments, specially biliverdin, are reported to be strongly photodynamic and induce genotoxicity whereas anti-mutagenic and anti-oxidative stress properties are described as well^16–18^. With these notions, the green pigments of *Bonellia* and *Chaetopterus* already began receiving particular attention due to their properties. It has long been suggested that bonellin, for instance, which is highly toxic against eukaryote and prokaryote microorganisms, is a potent natural anti-foulant responsible for keeping the sedentary worm *Bonellia viridis* free of biofouling^19^. Altogether, the state-of-the-art indicates that marine animal porphyrins can be highly diversified and that their function and biochemistry remain largely unknown.

The polychaete *Eulalia viridis* is an abundant species in Atlantic rocky shores, particularly noticeable due to its bright green coloration, which can dismiss its role in camouflage. It was previously noted that *Eulalia* does not possess complex chromatophores and owes its bright-green colour to specialised cells distributed along the skin that store the pigments in non-fluorescent cytoplasmic granules^20^. The distribution of pigment cells in the species is similar to what would be expected for melanins, for which no evidence was found asides nerve cord and eyes^21,22^. In line with the interest in porphyrins as potential photosensitisers and their physiological role as protective pigments, the aims of this study are: i) to evaluate the diversity and distribution of the pigments along *E. viridis* body, ii) to infer their basic spectroscopic properties and stability and iii) to contribute to understand the pigments’ adaptative value and their main functions.

## Results

### Pigment distribution

**Figure 1:**
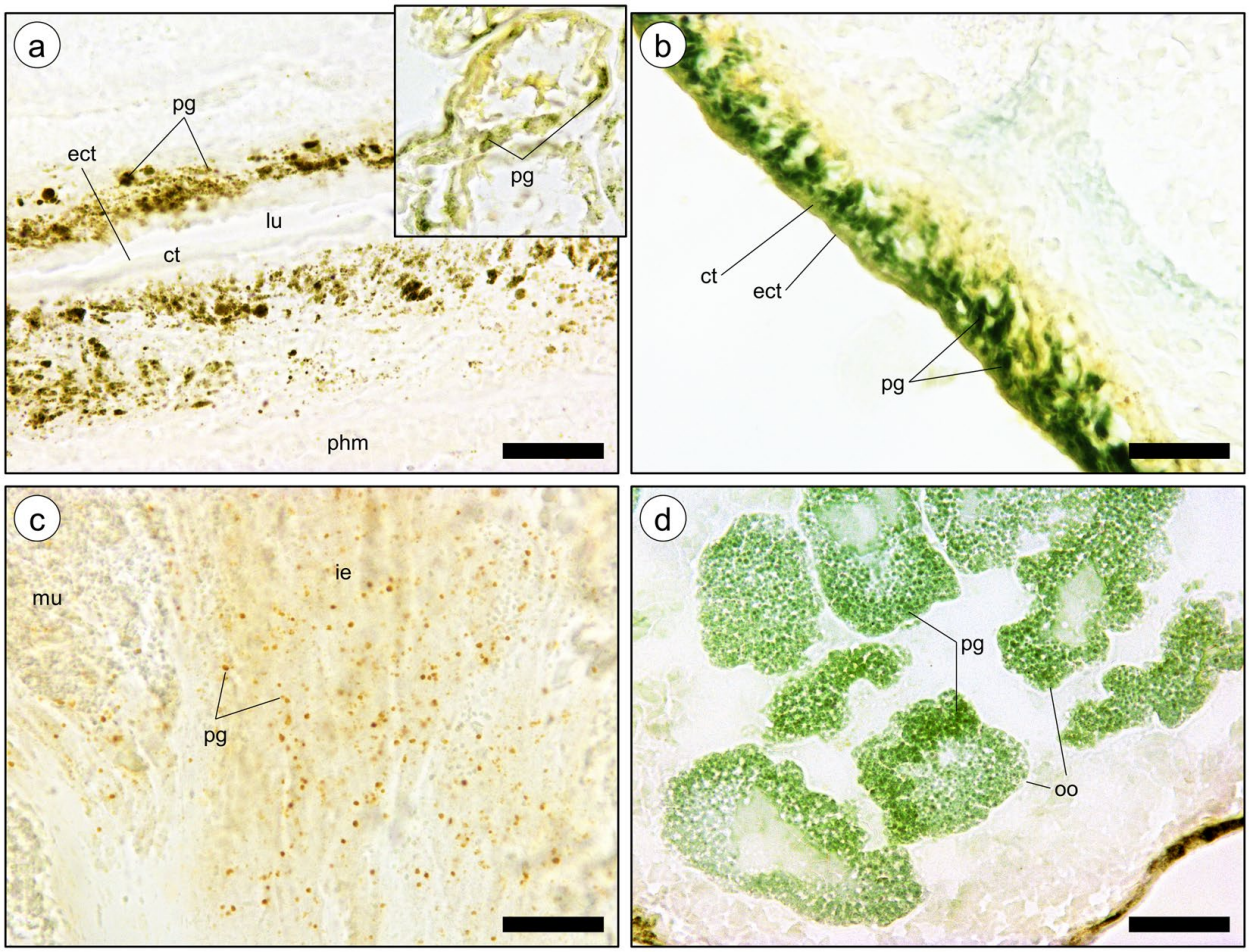
Frozen sections of various *E. viridis* organs (unfixed and unstained) to the disposition of revealing pigment granules. (**a**) Epithelium of the proboscis (inner lining) showing its pigment cells with pigment granules (pg). These cells are compressed between serous cells. ct, cuticle; ect, epicuticle; lu, lumen; phm, pharygeal musculature. Inset: Detail of pigment granules (pg) in sensorial papilla (outer lining of the organ). (**b**) Pigment granules (pg) inside pigment cells of the epidermis (skin), interleaved with other types, such as cuticle and mucus-secreting cells. ct, cuticle; ect, epicuticle. (**c**) Detail of pigment granules (pg) present in the intestine, exhibiting a brownish colour. The tint has been found connected with the digestion phase^21^. (**d**) Oocytes (oo) showing clearly the green pigment granules (pg) in ooplasm. Scale bars: 50 µm.

**Figure 2:**
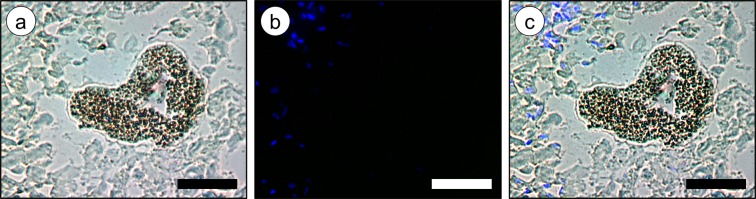
Micrograph of an *Eulalia viridis* oocyte (unfixed frozen section). (**a**) Micrograph showing the naturally-green pigment granules in brightfield. (**b**) The same section under ultraviolet (UV) light, evidencing only DAPI-stained nuclei (blue), whereas granules are devoid of fluorescence. (**c**) Merged image of (**a**) and (**b**). Scale bars: 50 µm.

Analysis of unstained frozen sections revealed that pigment granules tend to be naturally greenish, regardless of organ (Fig. 1). However, there was considerable inter-organ variation regarding distribution and colour range of pigments. Granules in pigment cells of the proboscis, in both internal and external epithelia, were more distinctively yellow-brownish (Fig. 1a and respective inset). On its turn, the skin epithelium had a dense layer of clear green pigments, caused by packed granules in single-layered pigment cells intercalating with mucocytes and other secretory cells that are devoid of noticeable pigmentation. The base of the epithelium yielded a conspicuous yellowish tint (Fig. 1b). The intestine (Fig. 1c) exhibited the broadest span of pigment colours, ranging from light green to brownish-orange. Pigments were allocated in granules and, seemingly, in endosomes and cytosols of the long epithelial (digestive) cells. Oocytes held bright-green granules that were scattered throughout the ooplasm. These were found from earlier stages of maturation and on (Fig. 1d). Pigment granules of any organ were not fluorescent under UV light, as shown in the example of Fig. 2.

### Pigment analysis by HPLC-DAD

#### Experiment A. Determining main pigments

Chromatograms were found to be complex, with multiple peaks with strong absorption in the UV and also in the violet or red regions of the visible spectrum. These results are consistent with the existence of both yellowish and greenish pigments in all organs, in accordance with visual inspection of extracts. Accordingly, three wavelengths were selected for detailed analysis: i) 280 nm to identify the absorption maxima in the UV zone; ii) 440 nm for yellowish pigments with maximum absorption in the range of violets, and iii) 700 nm for greenish pigments that absorb mostly in the red region. Individualisation of pigments was primarily based on retention times. Individualised spectra acquired by DAD were then analysed within each retention time to ascertain differences between pigments.

**Figure 3:**
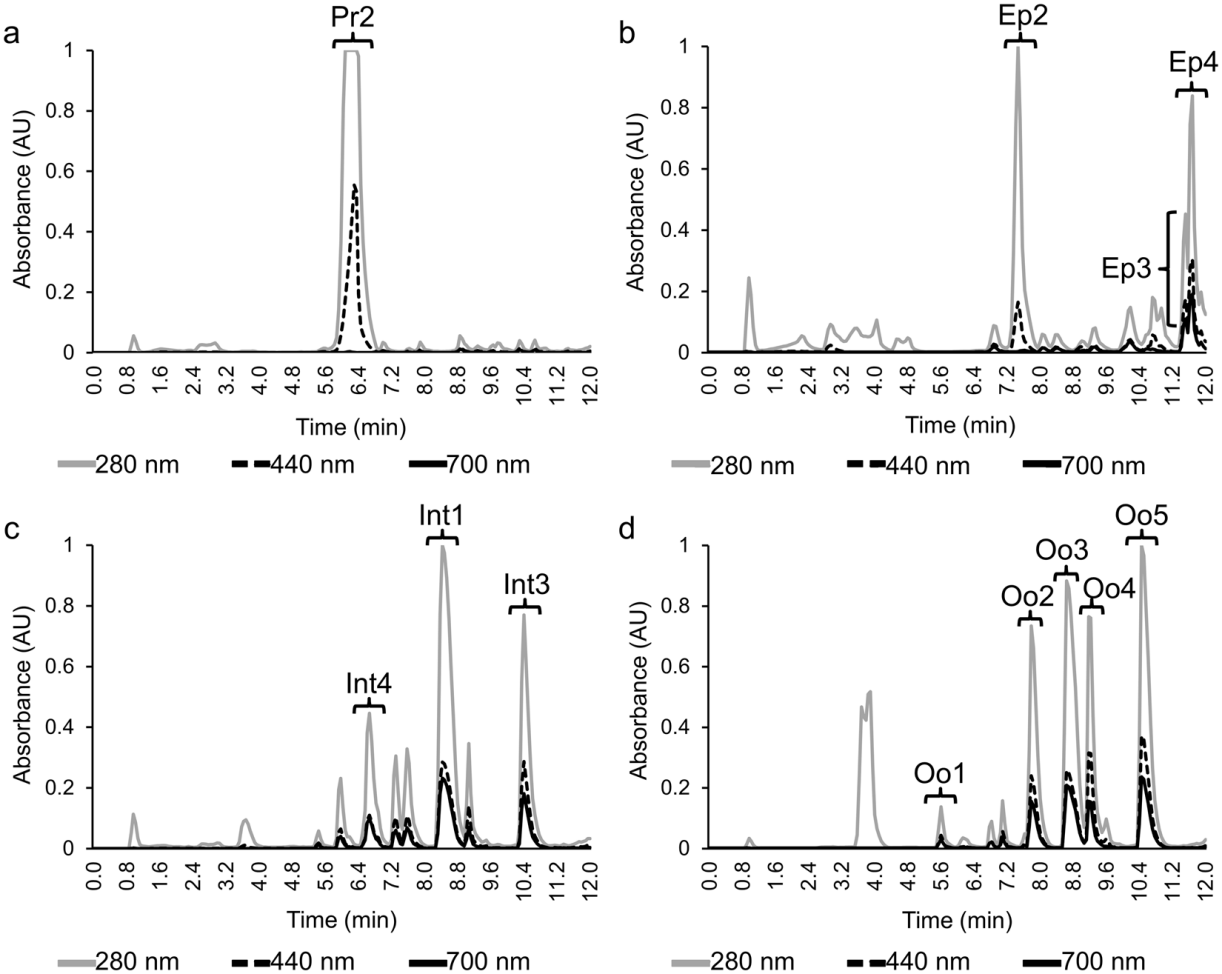
High-performance liquid chromatography with a detector diode array (HPLC-DAD) chromatograms from Experiment A (analysis of pigment absorption spectra). The main absorption maxima and retention times are illustrated for each *Eulalia viridis* organ. Absorbance data was collected for the wavelengths of 280, 440 and 700 nm, corresponding to absorbance in UV, violet (for yellow pigments detection) and red (for green pigments). Lettering identifies the major pigments. (**a**) Proboscis. (**b**) Epidermis. **(c**) Intestine. (**d**) Oocytes.

The pigments thus identified differed between organs (Table 1 - Experiment A). The proboscis yielded a yellowish pigment termed Pr2, which was detected at retention times between 6- and 6.5-min and presented maxima at both 280 nm and 440 nm (Fig. 3a). Another yellow pigment (Ep2) was detected in the epidermis (Fig. 3b), with higher retention time, but lower absorbance magnitude than the proboscis pigment. The chromatogram corresponding to extracts from epidermis (see Fig. 3b) also shows two greenish pigments (named Ep3 and Ep4), with retention times 11.3 and 11.8 min and high absorption at 280 nm and moderate absorption at 440 nm and 700 nm.

**Table 1:** Summary of the main pigment absorbance maxima registered in Experiments A (preliminary analysis of pigments), B (assessing interindividual variability) and C (determining stability through 6-week storage at −20 °C), plus their respective retention time (min).

	Pigment ID	Colour	Experiment A	Experiment B	Experiment C
Proboscis	Pr1	Yellow	X	3.2–3.7	3.4–3.7**
	Pr2	Yellow	6–6.5	7–7.4	7.1–7.6
Epidermis	Ep1	Yellow	X	3.4–3.7	3.4–3.7**
	Ep2	Yellow	7.3–7.8	7–7.4	7–7.4
	Ep3	Green	11.3–11.6	10.9–11.1	10.8–11.1
	Ep4	Green	11.6–11.8	11.1–11.6	11.1–11.5
Intestine	Int1	Green	8.3–8.8	9.5–10	9.4–9.9
	Int2	Green	X	10.8–11.2	X
	Int3	Green	10.3–10.7	11.3–11.6*	11.1–11.5
	Int4	Green	6.5–6.9	X	8.4–8.7
Oocytes	Oo1	Green	5.5–5.7	X	6.3–6.8***
	Oo2	Green	7.7–8.1	X	7.6–8***
	Oo3	Green	8.6–9	X	8.8–9.3***
	Oo4	Green	9–9.5	X	9.8–10.5
	Oo5	Green	10.4–10.9	X	11.2–11.7

*Int3 was not found in in one of the six samples.

**Pr1 and Ep1 were not found after the first week of storage.

***Oo1, Oo2 and Oo3 were found only after the first week of storage.

The intestine revealed three major green pigments termed Int1, Int3 and Int4, with retention times between 6.5 and 10.7 min (Fig. 3c). Similar to what was recorded in the epidermis and intestine, oocytes (Fig. 3d) revealed numerous pigments, with five being the most representative (Oo1, Oo2, Oo3, Oo4 and Oo5). These pigments had higher retention times, all being retrieved after 5.5 min. Pigments from both intestine and oocytes were greenish (refer to Table 1), revealing, however, high absorption at 280 nm and more intense relative maxima at 700 nm than the yellowish pigments previously indicated as Pr2 and Ep2 (proboscis and epidermis, respectively).

**Figure 4:**
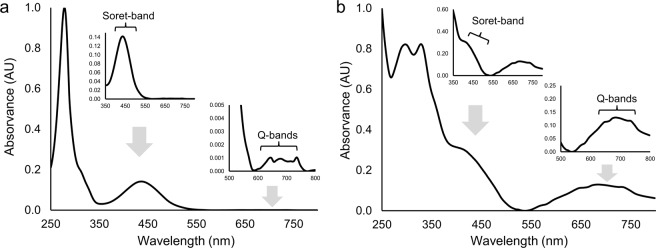
*Eulalia viridis* pigment absorption spectra retrieved from HPLC-DAD (Experiment A). (**a**) Yellow pigment (Pr1) from proboscis clearly showing Soret- (350–500 nm) and Q-bands (580–750 nm), characteristic of porphyrin pigments (*insets*). (**b**) Green pigment (Ep3) from epidermis also showing Soret- and Q-bands, exhibiting a shift of Q-bands toward higher wavelengths and less defined peaks (*insets*).

Absorption spectra revealed the Soret-band between 350 and 500 nm, followed by the characteristic four Q-bands (580–750 nm). These are clearly visible in yellow pigments from proboscis and epidermis (see example in Fig. 4a). However, in the absorption spectra of green pigments from epidermis, intestine and oocytes, both the Soret- and Q-bands are less defined (exemplified in Fig. 4b). In either case, the results reveal the porphyrinoid nature of pigments.

#### Experiment B. Assessing intraspecific variability

Experiment B, during which six independent pools of extracts were analysed, yielded, globally, the same pigments already described in Experiment A (Table 1 – Experiment B column). Nonetheless, additional yellow pigments were observed in both the proboscis (identified as Pr1) and epidermis (named Ep1), as revealed by distinct retention times and absorption maxima, but their representativeness was lower when compared with Pr2 and Ep2 mentioned earlier. An additional green pigment (Int2) was also observed between 10.8 and 11.2 min in intestine extracts. No extracts could be retrieved from oocytes during the course of this experiment, due to the unavailability of maturing females.

**Figure 5:**
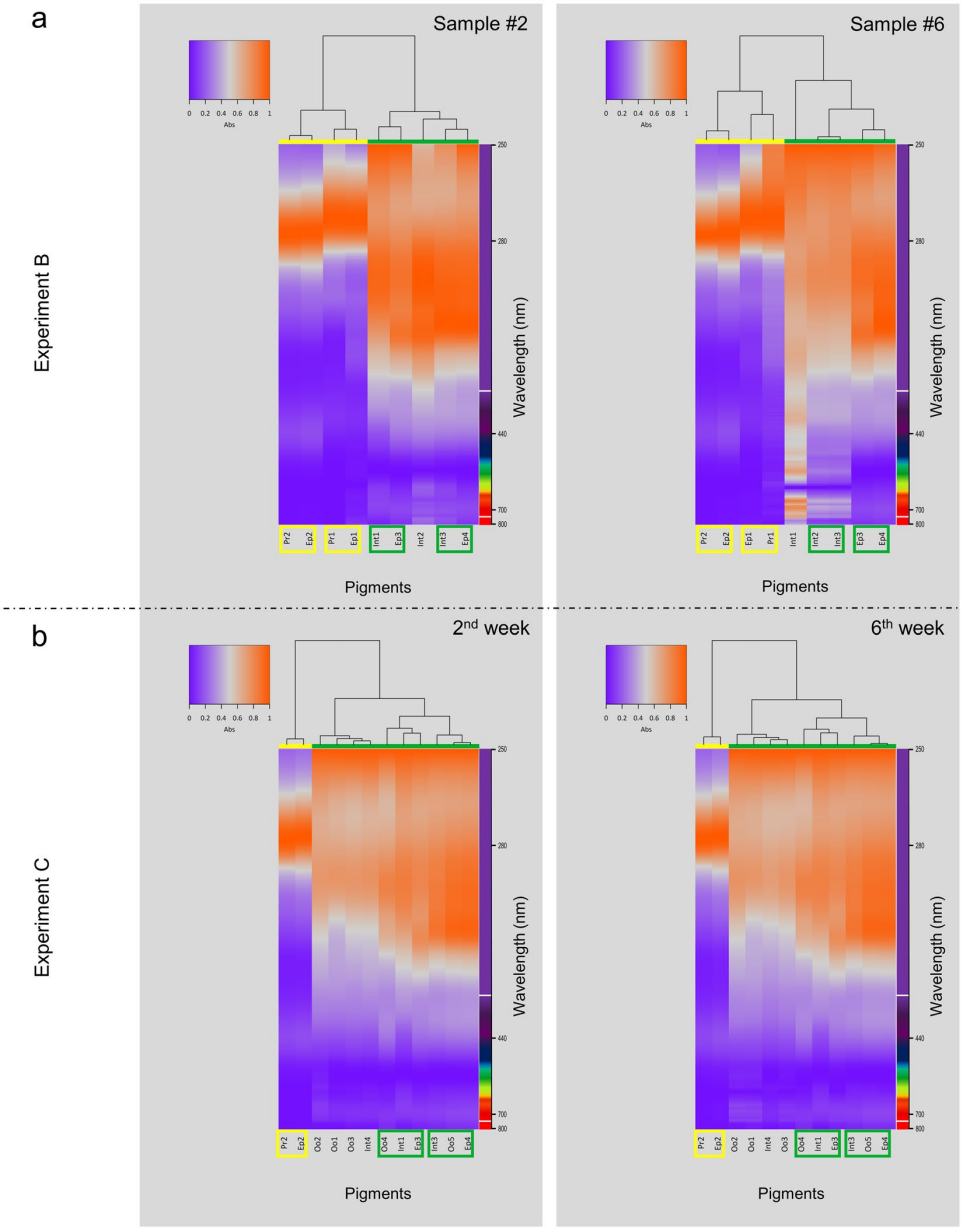
Heatmaps exemplifying the results from cluster analyses, evidencing associations between the two major types of pigments: yellow and green, highlighted by the colour bars below dendrograms. The clusters allocating the most important yellow and green pigments are highlighted by yellow and green boxes, respectively (refer to the text for further details). (**a**) Results from Experiment B, to assess interindividual variability, placing samples #2 and #6 side-by-side. (**b**) Results from Experiment C, comparing, as examples, weeks two and six of storage at −20 °C. Dendrograms are based on Euclidean distances.

Overall, pigments were divided in two major clusters, the leftmost allocating the yellow and the rightmost the greenish, as indicated by the colour bars in Fig. 5. Cluster analysis revealed similarities between pigments in proboscis, epidermis and intestine (exemplified in Fig. 5a). The yellow pigments found in the proboscis (Pr2) and epidermis (Ep2) had identical retention times (7 to 7.4 min) and the same absorption spectrum, which resulted in their allocation within the same clusters (even though absorbance was higher in Pr2). The same scenario was observed for the yellow pigments Pr1 and Ep1, from proboscis and epidermis, respectively. The associations between these yellow pigments remained unaltered among the six samples (upper dendrogram), therefore indicating reduced intraspecific variability. Similarities were also found among greenish pigments, albeit with moderately different retention times (Table 1), in both epidermis and intestine. Consequently, clustering between greenish pigments was not entirely consistent between samples, albeit the trend to associate pigments from the same organ. This is exemplified by the Int3 and Ep4 clustering and also by Int2 and Ep3, which, nonetheless, were found in only two of the samples (see dendrogram for sample #2 in Fig. 5a for an example). In the remaining samples, Int3 and Int2 were found associated, similarly to Ep4 and Ep3 (as for sample #6 in Fig. 5a).

#### Experiment C. Assessing pigment stability

As previously, a major division between yellow and green pigments was observed (Fig. 5b). Only scant variation between clusters of pigments was noted along the duration of the experiments, mostly due to an increase in individual pigments with storage time (refer to Fig. 5b as example). The yellow pigments of proboscis (Pr1) and epidermis (Ep1), detected in Experiment B, were also observed in Experiment C between 3.4 and 3.7 min, but only in the 1-week-old sample. The pigments from oocytes also presented similarities to those recorded earlier (i.e. during Experiment A). Contrary to Pr1 and Ep1, oocyte pigments Oo1 trough Oo3 were not detected in week 1 but in all subsequent weeks which may indicate that these are by-products of naturally-unstable intermediate forms. Cluster analysis also revealed association between the yellow pigments from the proboscis (Pr2) and epidermis (Ep2), which are clearly separated from the remaining pigments (see upper dendrograms in Fig. 5b). This experiment showed an association between green pigments that were consistently clustered and present in epidermis, intestine and oocytes along the six weeks of the experiment. Among green pigments the two rightmost subclusters in the dendrogram of Fig. 5b, consistently associated pigments from epidermis, intestine and oocytes, resulting in two groups, one allocating Ep3, Int1 and Oo4, and the other Ep4, Int3 and Oo5, all bearing retention times near 11 min.

**Figure 6:**
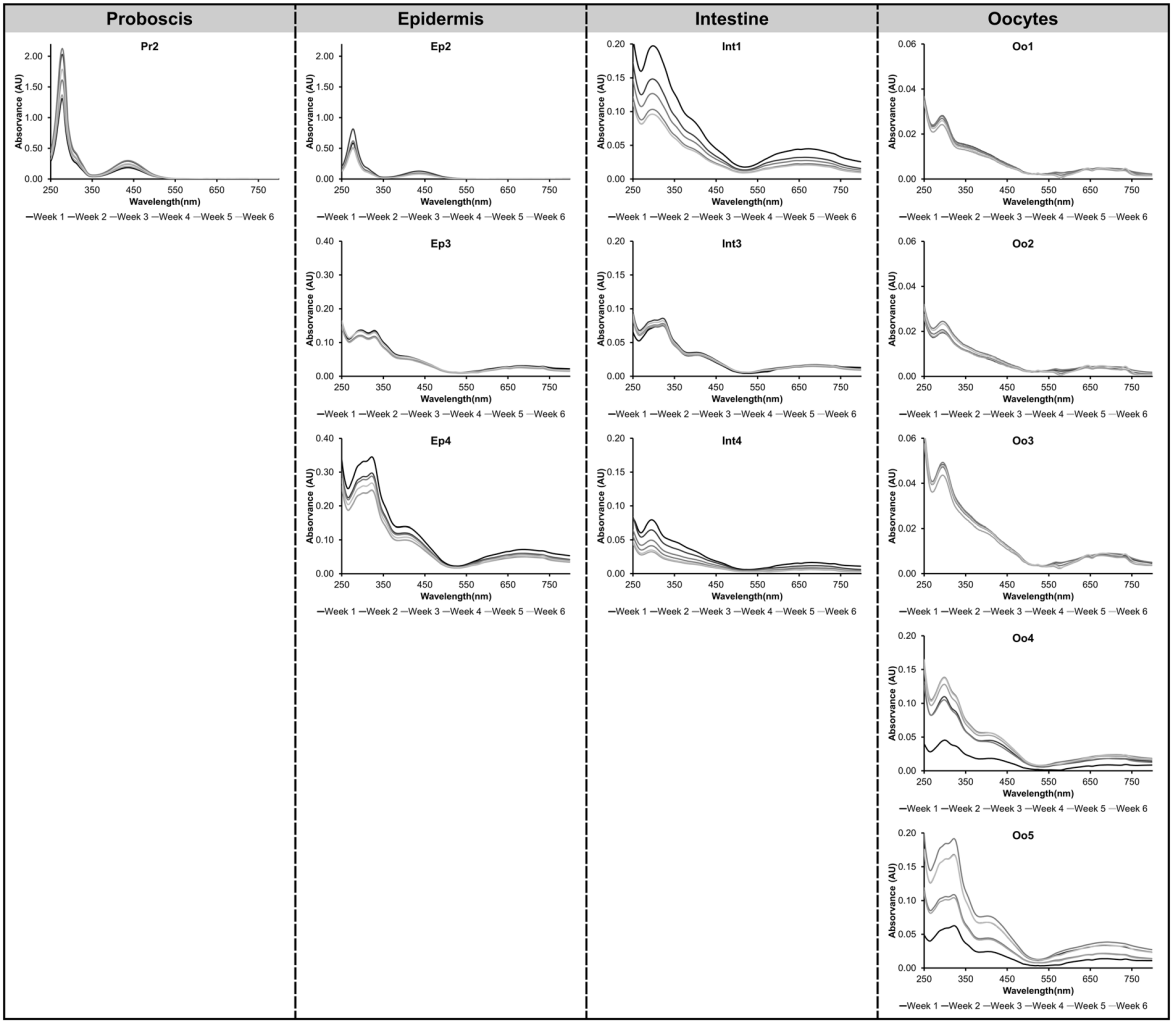
*Eulalia viridis* pigment absorption spectra collected during analysis with HPLC-DAD from Experiment C (addressing the stability of pigments) in proboscis, epidermis, intestine and oocytes along the six weeks of storage at −20 °C.

The yellow pigments found in the proboscis (Pr2) and epidermis (Ep2) showed highest stability during storage at −20 °C, without substantial alterations in absorbance maxima (Fig. 6). The green pigment Ep3, retrieved from the epidermis’ extract, also remained stable. However, a similar pigment, Ep4, yielded a small, but gradual decrease in absorbance over the six weeks of storage. The same gradual decrease was also verified for green pigments from the intestine, except Int3, which remained unaltered seemingly over time. Most pigments from oocytes remained stable over time, with the exception of Oo4 and Oo5, albeit without a clear trend, as shown in Fig. 6.

## Discussion

The bright green colour of *Eulalia viridis* results from the combination of multiple greenish and yellowish pigments that absorb strongly within the UV range and do not exhibit fluorescence in their native state. The current findings show that the worm’s major organs have their own pigment signatures. Greenish pigments are found in every organ, whereas yellowish pigments are mostly circumscribed to skin and, moreover, the proboscis. It is, in fact, in the skin where specialised pigment cells are more abundant, followed by, although in lower numbers, in the proboscis, namely within the pharynx epithelium and in the sensorial papillae (recall Fig. 1). Green pigments were also found in intestine and oocytes, even though the first case is devoid of specialised cells. In these latter situations, yellowish pigments seem to be almost absent. Altogether, there is a wider range of green pigments (i.e. with a major contribution of absorption maxima around 700 nm), compared to yellow (maximum at 440 nm). Conversely, the two major yellow pigments here described (from the proboscis and epidermis) seem to be either the same compound or closely related, whereas they appear to be very significantly distinct from all the green pigments. In addition to the seeming higher variety of green pigments, these exhibit higher interindividual variability as well, comparatively to yellow pigments. However, there are obvious similarities between the major green pigments from intestine, epidermis and oocytes, which were also found to be relatively stable after periods of storage in freezing conditions. Altogether, the findings indicate that yellow and green pigments are chemically distinct.

The pigments in *Eulalia* are chiefly stored in the form of intraplasmatic granules (recall Fig. 1). The absence of melanophores in the surface of the animal leads to reasoning that porphyrin-like pigments replace melanins in the role of protection against UV light, which is critical for a soft-bodied dweller of the intertidal. Colourful pigmentation in errant Polychaeta of rocky shores is far from uncommon. Indeed, the distribution of cells and pigment granules is similar to what is reported for biliverdin from *Hediste* (*Nereis*) *diversicolor*, for instance, another intertidal predatorial annelid^7^. Pigments in polychaetes, however, can have a wide variety of functions, from protection against light, UV and parasites to camouflage, even though the subject is far from understood. In the specific case of *Eulalia*, the purpose that may be swiftly exclude is camouflage, since the bright green colour does not significantly provide camouflage in the intertidal rocky shore. Looking into the spectra of *E. viridis* pigments, it is clear that there is strong absorption of UV light, regardless of major pigment type, yellow or green, or organ (recall Fig. 6), even though it is not naturally UV-fluorescent, similarly to melanins. On the other hand, the role in photoreception of integumentary pigments from some marine invertebrates is long known, at least since the studies on a holothurian (*Holothuria parvula*), whose greenish-yellow skin pigments are believed to play a key role in the animal’s photophobic behaviour^23^. In fact, *E. viridis* tends to avoid light by actively seeking shelter under rocks and mussels and the ideal time of collection has been found to be during early dawn or dusk (own observations). In either case, it is suggested that the pigments from *E. viridis* hold a dual function in protecting the animal from sunlight. Besides photoreception, green pigments can take part in other sensory function, as they are present in the mechanoreceptors in the papillae of the proboscis, and in the pigment cells of skin, the latter of which possess cilia that protrude through the cuticle, being hypothesised to have a sensory-like function^22^.

Porphyrin-like pigments are also believed to have an important function in the protection of egg masses of marine invertebrates. The females of *Phyllodoce mucosa* secrete a greenish mucus, resulting from the presence of biliverdin, with which they cover the fertilised eggs when they are laid on the bare surface of intertidal sandflats, where solar radiation, especially within the UV range, induces formation of reactive oxygen species, therefore offering antioxidant defence^24^. It can be inferred that the biliverdin-like pigments within the eggs of *E. viridis*, whose egg masses in the rocky intertidal are overlaid with unpigmented mucus, may have the same protective function against daylight. Indeed, the antioxidant properties of biliverdin and bilirubin are well-known, as they efficiently scavenge oxidative radicals, with oxidised bilirubin being recycled back into biliverdin via biliverdin reductase^25,26^. It must be noticed that mucus itself has been found to have antimicrobial properties in several other polychaetes, even though such has not been linked to the presence of pigments^27^. Still, one of the best-known marine animal porphyrins, bonellin, is active against both eukaryotes and prokaryotes and has been suggested to play an important role in *Bonellia* as chemical defence against foulants and eventual predators^19,28^, which suggests a potential role of porphyrinoids as natural biocides.

The pathways of biotransformation and catalysis of porphyrins in marine invertebrates, the Polychaeta in particular, are not understood, as well as their translocation and excretion. It has been disclosed that haemoglobin breakdown in *H. diversicolor* and subsequence transformation of haem to biliverdin, occurred close to blood vessels in the body-wall and proboscis, with the pigments being afterward either stored in epidermal cells or transported by coelomocytes to the intestine, where they are finally degraded and excreted^7^. An identical process is most likely to occur in *E. viridis*. Also, the coelomocytes are long known to be involved in nutrient transfer to oocytes during vitellogenesis^29^, which may explain also the prolific presence of pigment granules in germ cells. Furthermore, the intestine in *E. viridis* is clearly involved in pigment metabolism as well^21^. In fact, this function for the intestine can explain higher variety of pigments in this organ, compared to proboscis, skin, and oocytes. On the other hand, the existence of a few common pigments between these organs indicates translocation (recall Fig. 5a). Thus, the green and yellow pigments found in both the epidermis and proboscis seem to be a more final and stable form, as demonstrated by Experiment C.

The overall good stability, after archiving at −20 °C, of major yellow and green pigments from all organs, especially proboscis and epidermis, opens the door for possible applications of these bile-like pigments, a class of substances that is known to be powerfully photodynamic^16^, therefore showing potential for biomedical purposes, e.g. as photosensitisers in photodynamic therapy^15^. In fact, in the cases where true endogenous green pigments have been identified in the Polychaeta, tetrapyrroles were consistently present^4,5^. It must be noticed, though, that the origin of invertebrate tetrapyrroles is diverse. For instance, in *Chaetopterus variopedatus* the mid-gut pigment termed chaetopterin, which is a blend of breakdown products of chlorophyll (especially pheophorbides *a* and *b*), is connected with their feeding behaviour as detritivores^6^. On the other hand, in *B. viridis*, the green pigment bonellin is described as a chlorin resulting from protoporphyrin degradation^9,30^. In the case of *Eulalia*, the present findings give an indication of haem by-products, since when analysing the absorbance spectra of pigments (Fig. 6), no resemblance with chlorophyll or its derivatives (such as pheophorbides) was noticed^31^, nor the typical fluorescence of chlorophylls^32,33^. It must be noticed, though, that the acidic nature of the extraction solvent (comprised of HCl and acetonitrile) and elution buffer (phosphate buffer pH 3.5 and methanol) likely contributed to the stability of the pigments, by preventing, at least partly, agglomeration, in accordance with recent studies. Zannotti *et al*.^34^, verified this effect in a porphine in water-ethanol mixtures, highlighting as well that increased proportions of organic solvents induced agglomeration. The same authors analysed the spectra using multiple techniques, namely UV-vis spectroscopy, fluorescence and Raman, concluding that aggregation induces important spectral changes, including the masking of Soret- and Q-bands typical of porphyrins. This information aids explaining less-defined Soret- and, moreover, Q-bands in some of the pigments (Fig. 4). Altogether, the findings highlight the importance of enforcing consistent methods for the extraction and elution of pigments, as well as constraints to provide comprehensive characterisation of novel porphyrinoids.

Our findings support the original report by MacMunn^35^, who had already excluded the chlorophylloid nature of *Eulalia* green pigments, without, however, being able to trace their chemical nature. Altogether, the endogenous porphyrinoid pigments of *E. viridis* appear to be similar in function and origin to the coloured bile pigments of *H. diversicolor*. It must be noted that biliverdin in this latter species is described as a secondary pigment, since the green coloration is only noticeable when the presence of the worm’s main pigments, namely carotenoids, decline during the reproductive phase and/or in starving individuals^7^. However, in *E. viridis*, green pigments are unequivocally predominant, likely being homologous to biliverdin, whereas yellow pigments may be more similar to another product of haem breakdown known as bilirubin (also yellow), rather than carotenoids. In fact, the yellow pigments found in the proboscis and epidermis (see Fig. 2) have quite distinct spectra from carotenoids^36^. The assumption of pigments analogous to bilirubin and biliverdin as the main pigments present in *E. viridis* is supported by the analysis of bile pigments in humans, since the absorption spectra show great similarities, especially the maximum absorption peaks at 400 and 700 nm here found for the green pigments^37^.

## Conclusions

Animal pigments hold key adaptive value, playing key roles in their physiology and ecology. The uncanny bright-green pigmentation of the Polychaeta *Eulalia viridis* is evolutionarily designed as a means to thrive in the rocky intertidal, where it has no known major predators and furthermore, must deal with periods of exposure to daylight, as it is a diurnal opportunistic scavenger and predator of other invertebrates, especially bivalves and barnacles. The species are endogenous and seemingly haem-derived. They replace melanins in most of their body plan, being present, inclusively, in the ooplasm, and can be divided in two major types, each owning a distinct tint, yellow and green, the former being clearly predominant in the proboscis. There is, however, higher variety of green pigments, especially in the intestine, which is likely the most involved in final degradation and disposal. The pigments are, in most part, stable after storage under freezing conditions, which brings good prospects for further studies, given the novelty of these pigments and the potential of porphyrins and photosensitisers. Altogether, their function in UV protection, antimicrobial and possibly even sensorial offer this relatively simple organism effective solutions for adaptive success in the rocky intertidal.

## Materials and Methods

### Animal collection

*Eulalia viridis* (≈120 mm total length and weighting ≈250 mg each) were collected from the intertidal rocky shore in Parede beach, West of Portugal (38°41′42″N; 09°21′36″W). Individuals were kept in a mesocosm environment recreating their natural habitat, consisting of dark-walled glass aquaria equipped with constant aeration and recirculation, and fitted with natural rocks and clumps of mussels (all collected from the same area) to provide shelter and feed, as set-up in previous studies with this species^21^. Salinity, temperature and photoperiod were restrained within 35, 16 °C and 12:12 h, respectively.

### Choice of target organs and microscopy analysis

Epidermis (Ep), proboscis (Pr), intestine (Int) and oocytes (Oo) were chosen as target organs in the onset of previous works on *Eulalia viridis*^21,22^.These works revealed specialised pigments cells in epidermis and proboscis, as well as scattered pigment granules in epithelial cells of the gut and ooplasm. In the present work, cryopreservation and cryotomy were enforced to evaluate the pigments’ native appearance, i.e., without interference of fixatives and other chemicals used in histological and cytological processing. For the purpose, *E. viridis* were snap-frozen in liquid nitrogen, sectioned and placed in optimal cutting temperature (OCT) medium in an appropriate tissue mould. The OCT medium containing the tissue was frozen to have a solid support which allowed to cut longitudinal sections with 5–15 μm thick in the cryostat at −20 °C in a cryomacrotome (CM3600 XP, Leica Biosystems). Sections were transferred to pre-adhesivated slides (Thermo Scientific Superfrost Ultra Plus) and stored at −80 °C until observation in a DM 2500 LED model microscope equipped with a MC 190 HD camera (both from Leica). Both brightfield and autofluorescence observations were made. Samples were marked with Hoechst 33258 fluorochrome for DNA counterstaining.

### Pigment extraction

The extraction of pigments was performed consistently among all experiments to enable comparative assessment. *Eulalia viridis* were microdissected and the target organs separated. Oocytes (Oo) were obtained from maturing females. Porphyrin-like pigments were obtained according to the protocol developed by Woods and Simmonds^38^, with several modifications. In brief: Samples were then homogenised in one volume of a mixture of 1 N hydrochloric acid and concentrated acetonitrile (1:1) and subsequently centrifuged for 10 min at 10 000 g. The supernatant (fraction containing the pigment) was collected and stored at −20 °C until subsequent analyses. Freezing was found to assist the removal of residual aqueous phase and precipitates. Pigment extraction was performed in a darkened environment. Exposure of samples and extracts to air was kept to the minimum.

### Pigment chromatography

The organic phase of pigment-bearing extracts from each of the four organs (proboscis, epidermis, intestine and oocytes) were filtered with a GHP filter before analysis. High-performance liquid chromatography (HPLC) was based on the protocol for separation and quantification of porphyrins developed by Woods and Simmonds^38^, with many optimisations. Pigment analysis was carried out on a 100 × 10 mm i-d- Onyx Monolithic Semi-prep C-18 column, in a Merck Hitachi equipment fitted with a detector diode array (DAD). The solvents used for gradient elution were sodium phosphate buffer 10 mM, pH 3.5 (solvent A) and 100% (v/v) methanol (solvent B). After column stabilization with solvent B, 200 µl of sample per extract was injected. Elution was performed at a flow rate of 9 mL/min using a 10 min linear gradient from 65% to 35% of solvent A with an inverse gradient (from 35% to 65%) of solvent B, followed by isocratic elution at 95% of solvent B for 5 min. Throughout analysis the pressure was maintained at about 67 bar and the column temperature at 40 °C using a column heater.

### Experimental design

Three different experiments were performed, here forth denominated Experiment A, B and C. Experiment A was the first approach to the identification of the more representative pigments in extracts from each organ, according to spectral maxima per retention time. Experiments B and C were performed with the same conditions for extraction and HPLC-DAD but were designed to evaluate inter-individual variability and pigment stability, respectively, as described below.

#### Experiment A. Determining main pigments

The Experiment A involved analysis of individual pigment extracts per organ. Each of these four extracts was obtained from pooled individuals (five specimens), therefore allowing sufficient material for the first analytical screening albeit sacrificing inter-individual variability. The HPLC-DAD analyses were performed after 24 h of storage at −20 °C. The absorbance maxima for each organ were identified in chromatograms and the absorption spectrum or each pigment was recorded in accordance to retention time.

#### Experiment B. Assessing intraspecific variability

Experiment B aimed at assessing intraspecific variability. For the purpose, six independent extracts (samples) were obtained for each organ. Each sample consisted of a pool of five specimens. Samples were stored at −20 °C until analysis by HPLC-DAD, which was done in the same day to exclude technical variation.

#### Experiment C. Assessing pigment stability

Experiment C aimed at assessing the pigment stability over time. For the purpose, periods of storage at −20 °C, in the dark, were considered as paradigmatic conditions for archiving. In this case, a single extract per organ was obtained (pooling a total of 40 specimens to assure sufficient material). Each pooled extract per organ was analysed weekly by HPLC-DAD over the six weeks of experiment.

### Data analysis

The absorption spectra were analysed by hierarchical clustering using Euclidean distances as metrics and complete linkage as amalgamation rule for hierarchical clustering. Computations and heatmap generation was done with R 3.4.4^39^. Before analyses, all data was min-max normalized to values between 0 to 1 using the D-7000 HPLC System Manager software (Hitachi).
